# Association of Iron Homeostasis With One‐Year All‐Cause Mortality Among Intensive Care Unit Patients with Atrial Fibrillation: A Retrospective Study

**DOI:** 10.1002/clc.70494

**Published:** 2026-10-14

**Authors:** Mingze Wu, Juan Hua, Yao Zhou, Yu Zhang, Lin Zhang, Yu Wang, Qinmei Xiong

**Affiliations:** ^1^ Department of Cardiology The Second Affiliated Hospital, Nanchang University Nanchang China

**Keywords:** all‐cause mortality, atrial fibrillation, iron homeostasis‐related indicators, prognosis

## Abstract

**Background:**

Perturbations in iron homeostasis can substantially affect cardiovascular outcomes. This study aims to evaluate the prognostic value of various iron homeostasis indicators for all‐cause mortality in critically ill AF patients.

**Method:**

Data for this retrospective study were obtained from the Medical Information Mart for Intensive Care IV database. Based on the iron homeostasis‐related indicators (ferritin, serum iron, transferrin, and total iron‐binding capacity [TIBC]), the risks of 1‐year mortality among critically ill patients with atrial fibrillation were calculated using Cox proportional‐hazards regression. Associations of iron homeostasis indicators with all‐cause mortality in critically ill AF patients were assessed via Kaplan–Meier curves, restricted cubic splines, and subgroup analyses.

**Results:**

After multivariable adjustment, patients in the highest ferritin quartile had a greater risk of 1‐year mortality than those in the lowest quartile (Q4 HR 1.277, 95% CI 1.083−1.506, *p* < 0.01). In contrast, the highest quartiles of transferrin (Q4 HR 0.732, 95% CI 0.622−0.861, *p* < 0.01) and TIBC (Q4 HR 0.732, 95% CI 0.622−0.861, *p* < 0.01) were associated with lower mortality. No significant association was observed between serum iron quartiles and 1‐year mortality (Q4 HR 1.067, 95% CI 0.914−1.245, *p* = 0.41). Restricted cubic spline analyses further characterized the exposure and outcome patterns for these iron‐related parameters.

**Conclusion:**

Among critically ill patients with AF, ferritin, transferrin and TIBC levels were independently associated with 1‐year all‐cause mortality. No significant association was observed for serum iron.

## Graphical abstract

This retrospective study examined the prognostic role of iron markers (ferritin, serum iron, transferrin, TIBC) in intensive care unit patients with atrial fibrillation. Elevated ferritin was associated with increased mortality, whereas higher transferrin and TIBC predicted lower mortality, highlighting their potential for risk stratification.

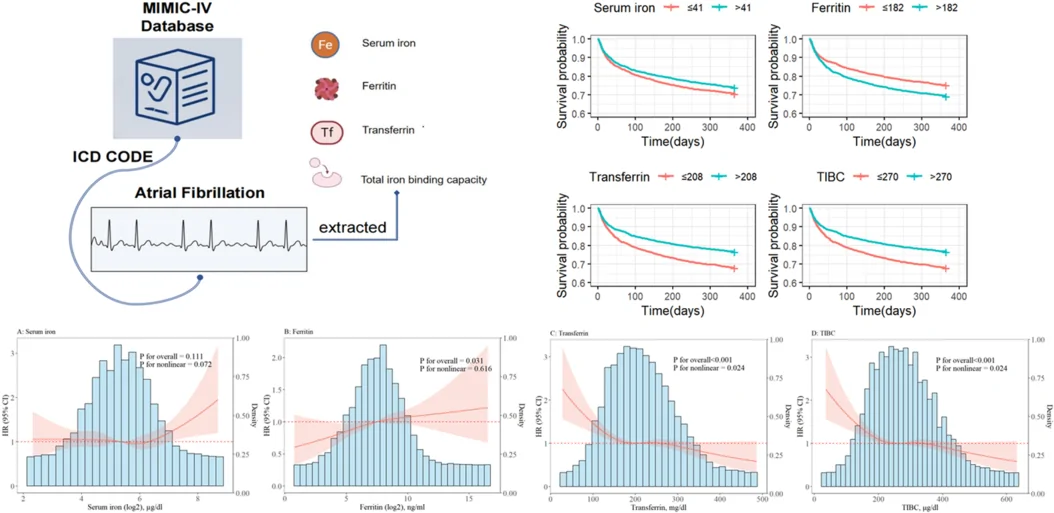

## 1 Introduction

Atrial fibrillation (AF) is a highly prevalent arrhythmic disorder characterized by increased incidence and poor survival outcomes [1]. The prevalence of AF showed a significant rising trend from 1990 to 2019, with about 59.7 million cases of AF worldwide, which was approximately doubled when compared to 1990 [2]. AF increases the risk of various severe complications, complicates clinical management, raises healthcare costs, and leads to elevated mortality [3]. Identifying these potential risk factors is crucial for enhancing the prognosis of critically ill patients with AF.

Iron is an essential element for the human body [4]. It is required for cellular survival, proliferation, and metabolism [5, 6]. Iron plays an essential role in oxygen transport and is involved in physiological processes such as immune function [7], ATP production [8], DNA replication or repair, and cell cycle progression as well [9]. In addition, iron homeostasis is closely associated with inflammation, stress, and body consumption.

In iron homeostasis, ferritin has recently attracted growing attention as a potential prognostic biomarker in inflammation, with levels mirroring the degree of acute and chronic inflammation in infectious, rheumatologic, hematologic, and cardiovascular disease [10]. Increased ferritin tends to correlate with worse outcomes in disease conditions [11]. By comparison, serum iron levels are influenced by various factors and have relatively poor specificity, and changes in serum iron do not reliably correspond to improved survival outcomes [12]. Elevated levels of transferrin and TIBC, key serological indicators of iron and nutritional status, may reflect protective regulation in the body [13, 14].

However, the association of different iron homeostasis‐related indicators with long‐term outcomes in critically ill patients with AF remains incompletely characterized. Accordingly, we examined the associations of ferritin, serum iron, transferrin, and TIBC with 1‐year all‐cause mortality and further assessed the shape and consistency of these associations.

## 2 Methods

### 2.1 Source of Data

The data for this retrospective study were sourced from the Medical Information Mart for Intensive Care IV (MIMIC‐IV, version 3.1). Patient data were collected from the Beth Israel Deaconess Medical Center (BIDMC, Boston, MA, USA) from 2008 to 2022 [15]. Prior to data extraction, the first author, Mingze Wu, had been granted access to the database (certification number: 65951130). The institutional review boards of BIDMC and MIT both approved the ethical use of MIMIC‐IV data. All personal data were de‐identified, and informed consent was not required.

### 2.2 Study Design and Population

AF patients on their first intensive care unit (ICU) admission during their first hospitalization were included in this retrospective study. A total of 20 440 AF cases were identified using diagnostic codes from the International Classification of Diseases (ICD‑9 and ICD‑10). The exclusion criteria were as follows: (1) ICU stay duration < 24 h. (2) repeated ICU admissions for AF (only the initial admission was retained). (3) missing or incomplete records for key iron homeostasis markers (serum iron, ferritin, transferrin, or TIBC). (4) presence of liver disease (any severity), malignant cancer, metastatic solid tumors, or AIDS.

Ultimately, 4949 patients were included in the present study cohort and divided into four groups according to the quartiles of various iron homeostasis indicators (serum iron, ferritin, transferrin, and total iron‐binding capacity). The flowchart for patient screening is shown in the figure below (Figure 1).

**Figure 1:**
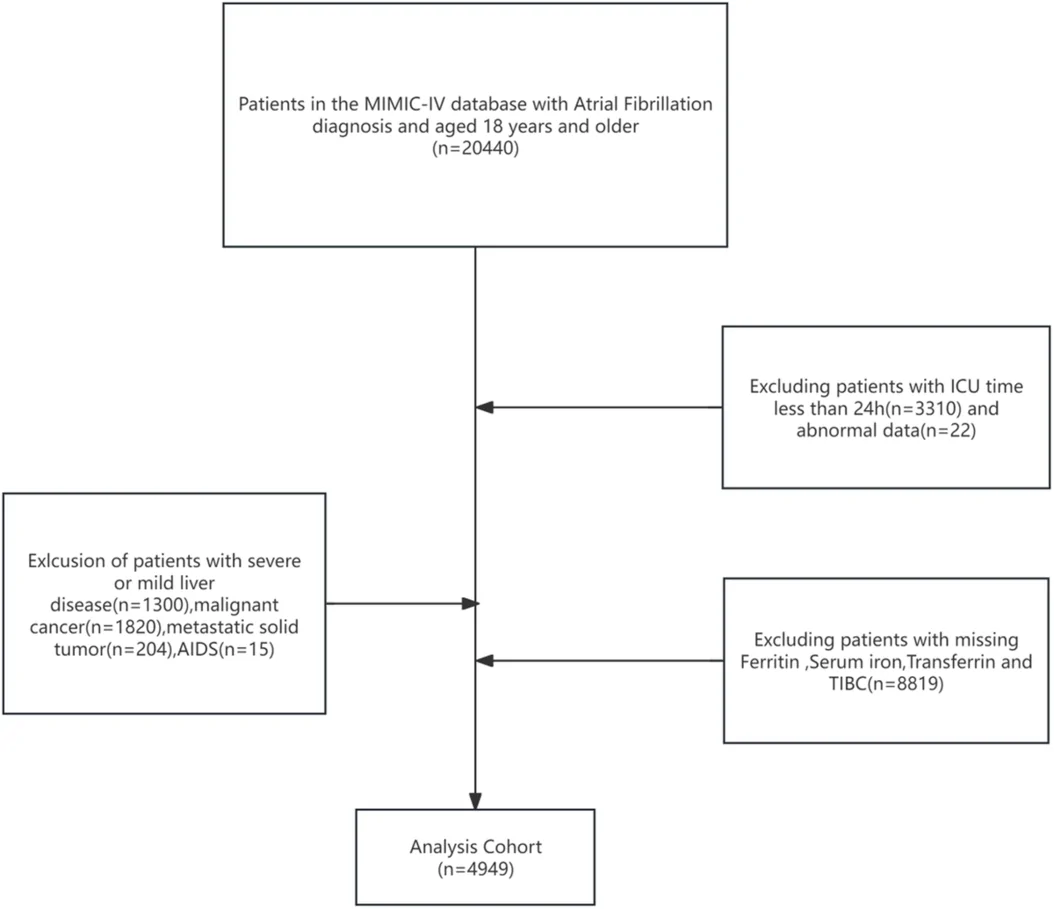
Flowchart for patient screening.

### 2.3 Data Extraction

Data extraction was performed using Navicat Premium (Version 16.1.15) with Structured Query Language (SQL) queries. The extracted potential variables were as follows: demographics (age, gender, race/ethnicity, weight), comorbidities (myocardial infarction, hypertension, diabetes, chronic obstructive pulmonary disease, congestive heart failure, peripheral vascular disease, cerebrovascular disease, dementia, rheumatic diseases), vital signs (systolic blood pressure [SBP], diastolic blood pressure [DBP], mean blood pressure [MBP], heart rate [HR], respiratory rate [RR], temperature), laboratory data (SpO_2_, pH, base excess [BE], anion gap, bicarbonate, glucose, blood urea nitrogen [BUN], creatinine, calcium, chloride, sodium, potassium, prothrombin time [PT], partial‐thromboplastin time [PTT], hemoglobin, platelets, white blood cells [WBC]), medication (aspirin, clopidogrel, calcium channel blocker [CCB], angiotensin‐converting enzyme inhibitors/angiotensin receptor blockers [ACEI/ARB], digoxin, diuretics, amiodarone, beta blockers, statin, novel oral anticoagulants [NOACs], heparin, warfarin), Glasgow Coma Scale [GCS], Simplified Acute Physiology Score II [SAPS II], Sequential Organ Failure Assessment [SOFA]), and length of stay (LOS in hospital and LOS in ICU).

Survival time was determined from the date of ICU admission using the recorded date of death. Baseline laboratory measurements and severity scores were defined using the first available values after ICU admission. Covariates with more than 20% missingness were not included in the analysis. For the remaining covariates, missing data were imputed using a random‐forest procedure implemented with the “mice” package in R software.

### 2.4 Outcomes

The primary endpoint for MIMIC‐IV database patients was defined as 1‐year mortality with at least 1‐year follow‐up.

## 3 Statistical Analysis

The normality of continuous variables was first examined using the Kolmogorov–Smirnov test. Normally distributed data were expressed as mean ± standard deviation (SD) and compared using Student's *t*‐test or one‐way ANOVA. For continuous variables not following a normal distribution, the Wilcoxon rank‐sum test was applied for group comparisons, and results were summarized as median (IQR). Categorical variables were compared using the chi‐square test or Fisher's exact test, as appropriate, and presented as frequencies with percentages.

Iron homeostasis indicators (ferritin, serum iron, transferrin, and total iron‐binding capacity [TIBC]) were stratified by median and quartiles. Kaplan−Meier (K‐M) curves were used to estimate survival distributions, which were compared using the log‐rank test. Univariate Cox analysis was adopted to assess the association between iron homeostasis indicators and 1‐year mortality. Variables with clinical significance or promising univariate outcomes were incorporated into the multivariate Cox proportional hazards model. The number of variables in the final model was determined with consideration of the number of outcome events. Model 1 was unadjusted. Model 2 adjusted for age, gender, race/ethnicity, while model 3 was adjusted for age, gender, race/ethnicity, weight, diabetes, hypertension, myocardial infarction, congestive heart failure, cerebrovascular disease, dementia, rheumatic disease, SBP, DBP, HR, RR, temperature, pulse blood oxygen saturation [SpO_2_], WBC count, hemoglobin, platelet count, anion gap, BUN, creatinine, blood glucose, calcium, chloride, potassium, international normalized ratio (INR), aspirin, clopidogrel, CCB, ACEI/ARB, digoxin, amiodarone, beta blockers, statin, NOACs, heparin, warfarin, GCS, SAPS II, and SOFA. In all three models, the lowest quartile of the iron homeostasis indicators was used as the reference. In this study, since ferritin and serum iron levels were substantially skewed, both variables were logarithmically transformed for continuous analysis.

Iron homeostasis‐related indicators were analyzed as continuous variables using restricted cubic splines (RCS) to explore their association with primary outcome risk. Subgroup analyses were performed for the following categories: gender, age (< 60/≥ 60 years), race/ethnicity, diabetes, hypertension, congestive heart failure, cerebrovascular disease, new‐onset AF, SAPS II, and SOFA.

All data analyses were conducted using R software (version 4.4.1). For all analyses, the level of *p* < 0.05 was considered statistically significant.

## 4 Results

### 4.1 Baseline Characteristics of Study Participants

This study extracted data from 20 440 patients in the MIMIC‐IV database. A total of 4949 patients with AF met the inclusion criteria and were included for analysis. The median age of participants was 73 years (IQR, 64−81), and 56.6% were male. We divided the patients into survivors and non‐survivors according to their 365‐day survival. Simplified baseline characteristics of the study cohort are listed in Table 1. Detailed baseline information is contained in Supporting Information S1: Table 1 (Additional file 1). Compared with survivors, non‐survivors were older and had higher ferritin levels, lower transferrin and TIBC levels, lower hemoglobin levels, poorer renal function (elevated BUN and creatinine), impaired coagulation (prolonged INR and PT), and higher disease severity scores (SAPS II and SOFA). Meanwhile, among patients who died within 1 year, the cumulative proportions of deaths occurring by 30, 60, 90, and 180 days were 38%, 54%, 64%, and 80%, respectively.

**Table 1:** Baseline characteristics of atrial fibrillation patients.

Characteristic	*N*	Overall *N* = 4949	Non survivors *N* = 1375	Survivors *N* = 3574	*p* value
Age	4949	73.00 (64.00, 81.00)	77.00 (70.00, 84.00)	71.00 (62.00, 79.00)	< 0.001
Gender, *n* (%)	4949				0.122
F		2150 (43)	622 (45)	1528 (43)	
M		2799 (57)	753 (55)	2046 (57)	
Race, *n* (%)	4949				0.096
Non‐White		1441 (29)	376 (27)	1065 (30)	
White		3508 (71)	999 (73)	2509 (70)	
Weight	4949	79.00 (66.40, 95.15)	74.20 (62.30, 89.40)	81.35 (68.00, 97.00)	< 0.001
Hypertension, *n* (%)	4949	3004 (61)	687 (50)	2317 (65)	< 0.001
Diabetes, *n* (%)	4949	1978 (40)	572 (42)	1406 (39)	0.155
Chronic pulmonary disease, *n* (%)	4949	1417 (29)	423 (31)	994 (28)	0.043
Myocardial infarction, *n* (%)	4949	1082 (22)	371 (27)	711 (20)	< 0.001
Congestive heart failure, *n* (%)	4949	2651 (54)	813 (59)	1838 (51)	< 0.001
Peripheral vascular disease, *n* (%)	4949	821 (17)	251 (18)	570 (16)	0.056
Cerebrovascular disease, *n* (%)	4949	637 (13)	219 (16)	418 (12)	< 0.001
Dementia, *n* (%)	4949	248 (5.0)	117 (8.5)	131 (3.7)	< 0.001
Ferritin, ng/mL	4949	182.00 (74.00, 427.00)	218.00 (93.00, 513.00)	170.00 (68.00, 390.00)	< 0.001
Iron, µg/dL	4949	41.00 (26.00, 64.00)	38.00 (25.00, 63.00)	41.50 (26.00, 64.00)	0.084
TIBC, µg/dL	4949	270.00 (208.00, 338.00)	250.00 (191.00, 320.00)	277.00 (215.00, 345.00)	< 0.001
Transferrin, mg/dL	4949	208.00 (160.00, 260.00)	192.00 (147.00, 246.00)	213.00 (165.00, 265.00)	< 0.001
LOS ICU, days	4,949	2.83 (1.70, 5.40)	3.46 (1.93, 7.67)	2.58 (1.60, 4.83)	< 0.001
LOS hospital, days	4949	9.66 (5.99, 16.65)	11.84 (6.60, 20.94)	8.99 (5.83, 15.09)	< 0.001
30 days death, *n* (%)	4949	529 (11)	529 (38)	0 (0)	
60 days death, *n* (%)	4949	741 (15)	741 (54)	0 (0)	
90 days death, *n* (%)	4949	874 (18)	874 (64)	0 (0)	
180 days death, *n* (%)	4949	1104 (22)	1104 (80)	0 (0)	

*Note:* Mortality at 30, 60, 90, and 180 days represents cumulative mortality by the corresponding time points after enrollment. SI conversion factors: to convert ferritin to μg/L, multiply by 1; serum iron to μmol/l, by 0.179; total iron‐binding capacity to μmol/L, by 0.179, transferrin to μmol/L, by 0.123. Data are presented as numbers and percentage or median and interquartile range.

Abbreviations: ICU, intensive care unit; LOS, length of stay; TIBC, total iron‐binding capacity.

### 4.2 Study Outcomes

#### 4.2.1 Kaplan‐Meier Analysis of 1‐Year Survival

Median‐based Kaplan‐Meier curves are presented in Figure 2. Patients with ferritin levels above the median had lower 365‐day survival than those with lower ferritin levels (log‐rank *p* < 0.01). In contrast, the above‐median groups for serum iron, transferrin, and TIBC showed higher unadjusted survival probabilities (all log‐rank *p* < 0.01). Quartile‐based analyses likewise showed significant survival differences across ferritin, transferrin, and TIBC categories, whereas no significant difference was observed across serum iron quartiles (Supporting Information S1: Figure 7).

**Figure 2:**
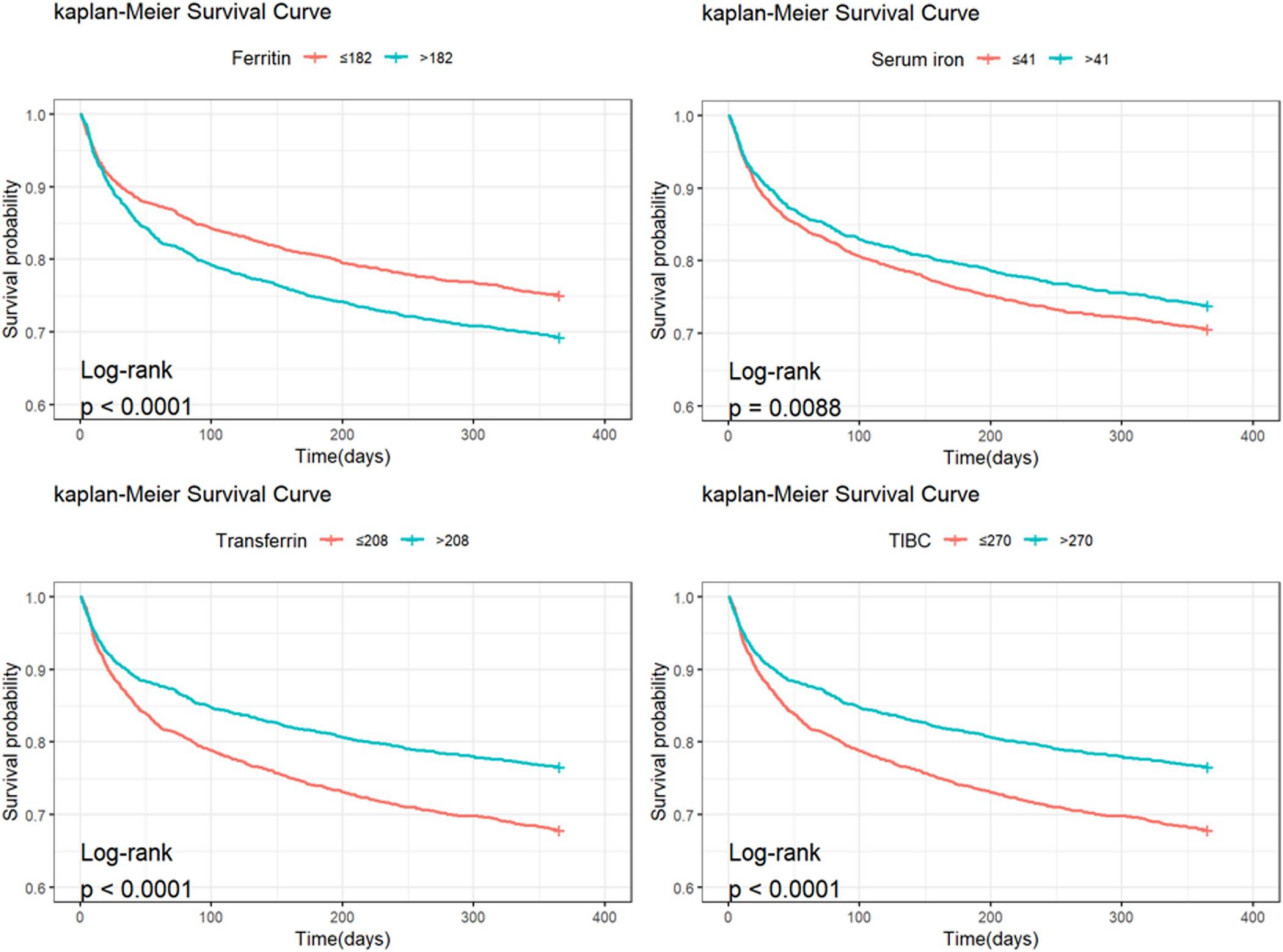
Kaplan−Meier estimates of 365‐day survival according to ferritin, serum iron, transferrin, and TIBC levels in critically ill patients with AF. TIBC, total iron‐binding capacity.

#### 4.2.2 Association between Iron Homeostasis and Mortality

In Model 3, the adjusted hazard ratio (HR) and 95% confidence interval (CI) for ferritin categories from low to high (≤ 74, > 74 to ≤ 182, > 182 to < 427, and ≥ 427) were 1.0 (reference), 1.237 (1.053−1.455), 1.258 (1.069−1.480), and 1.277 (1.083−1.506), respectively. For serum iron categories (≤ 26, > 26 to ≤ 41, > 41 to < 64, ≥ 64), the adjusted HR and 95% CI were 1.0 (reference), 1.046 (0.902−1.212), 0.919 (0.786−1.074), and 1.067 (0.914−1.245), respectively. For transferrin categories (≤ 160, > 160 to ≤ 208, > 208 to < 260, ≥ 260), the adjusted HR and 95% CI were 1.0 (reference), 0.838 (0.726−0.967), 0.819 (0.703–0.954), and 0.732 (0.622−0.861), respectively. For TIBC categories (≤ 208, > 208 to ≤ 270, > 270 to < 338, ≥ 338), the adjusted HR and 95% CI were 1.0 (reference), 0.838 (0.726−0.967), 0.818 (0.702−0.953), and 0.732 (0.622−0.861), respectively.

Across the three Cox models, the association between ferritin and mortality was most pronounced in the highest quartile, whereas serum iron showed no consistent association with 365‐day mortality. In contrast, the highest quartiles of transferrin and TIBC were associated with lower mortality relative to the reference quartiles. The results are shown in Table 2 and are also provided in Supporting Information S1: Table 2 (in Additional file 1).

**Table 2:** Cox proportional hazard ratios (HR) for 1‐year all‐cause mortality according to quartiles of iron homeostasis.

*Quartile*	Model 1			Model 2			Model 3		
	HR	95% CI	*p* value	HR	95% CI	*p* value	HR	95% CI	*p* value
*Ferritin*									
Q1	—	—	—	—	—	—	—	—	—
Q2	1.297	1.106‐1.521	0.001	1.325	1.129‐1.554	< 0.001	1.237	1.053‐1.455	0.010
Q3	1.361	1.162‐1.594	< 0.001	1.432	1.222‐1.679	< 0.001	1.258	1.069‐1.480	0.006
Q4	1.589	1.362‐1.853	< 0.001	1.823	1.559‐2.132	< 0.001	1.277	1.083‐1.506	0.004
*Iron*									
Q1	—	—	—	—	—	—	—	—	—
Q2	1.039	0.898‐1.203	0.604	1.016	0.878‐1.176	0.827	1.046	0.902‐1.212	0.555
Q3	0.857	0.735‐0.998	0.048	0.898	0.770‐1.046	0.167	0.919	0.786‐1.074	0.288
Q4	0.938	0.808‐1.089	0.398	1.013	0.872‐1.176	0.870	1.067	0.914‐1.245	0.413
*Transferrin*									
Q1	—	—	—	—	—	—	—	—	—
Q2	0.779	0.677‐0.896	< 0.001	0.749	0.651‐0.862	< 0.001	0.838	0.726‐0.967	0.015
Q3	0.666	0.576‐0.770	< 0.001	0.663	0.574‐0.767	< 0.001	0.819	0.703‐0.954	0.010
Q4	0.560	0.481‐0.652	< 0.001	0.579	0.497‐0.675	< 0.001	0.732	0.622‐0.861	< 0.001
*TIBC*									
Q1	—	—	—	—	—	—	—	—	—
Q2	0.780	0.678‐0.897	< 0.001	0.750	0.652‐0.863	< 0.001	0.838	0.726‐0.967	0.016
Q3	0.665	0.575‐0.769	< 0.001	0.662	0.573‐0.766	< 0.001	0.818	0.702‐0.953	0.010
Q4	0.561	0.482‐0.653	< 0.001	0.579	0.497‐0.675	< 0.001	0.732	0.622‐0.861	< 0.001

*Note:* Model 1: Unadjusted model. Model 2: adjusted for gender, age, race. Model 3: adjusted for gender, age, race, weight, diabetes, hypertension, myocardial infarction, congestive heart failure, cerebrovascular disease, dementia, rheumatic disease, systolic blood pressure, diastolic blood pressure, heart rate, resp rate, temperature, spo2, white blood count, hemoglobin, platelets, anion gap, bun, creatinine, glucose, calcium, chloride, potassium, INR, GCS, SAPS II, SOFA, Aspirin, Clopidogrel, Beta blocker, ACEI/ARB, CCB, Digoxin, Amiodarone, Statin, NOACs, Heparin, Warfarin.

Abbreviations: CI, confidence interval; HR, hazard ratio.

To validate the robustness of our findings, we first performed Bonferroni and FDR (Benjamini–Hochberg) corrections for multiple comparisons in the final multivariable model for iron homeostasis indicators; the statistically significant associations remained significant after correction (Supporting Information S1: Table 3, in Additional file 1). Second, similar estimates were obtained when the indicators were analyzed as median‐based categories or continuous variables. (Supporting Information S1: Table 4, in Additional file 1). Further adjustment for NLR (Neutrophil‐to‐Lymphocyte Ratio) and PLR (Platelet‐to‐Lymphocyte Ratio) indicators, along with exclusion of missing values, did not materially alter the overall pattern of results (Supporting Information S1: Table 5, in Additional file 1).

Furthermore, ferritin stratified by an iron overload threshold (SF > 1000 ng/mL) was significantly associated with 1‐year mortality in the univariate analysis, but the association was no longer statistically significant in the fully adjusted model (Supporting Information S1: Table 6, in Additional file 1).

#### 4.2.3 RCS Analysis

The RCS curves showed an approximately linear increase in 1‐year mortality risk with increasing log2‐transformed ferritin. Mortality risk declined as transferrin and TIBC increased, with nonlinear patterns observed for both markers. No statistically significant association was evident for log2‐transformed serum iron (Figure 3).

**Figure 3:**
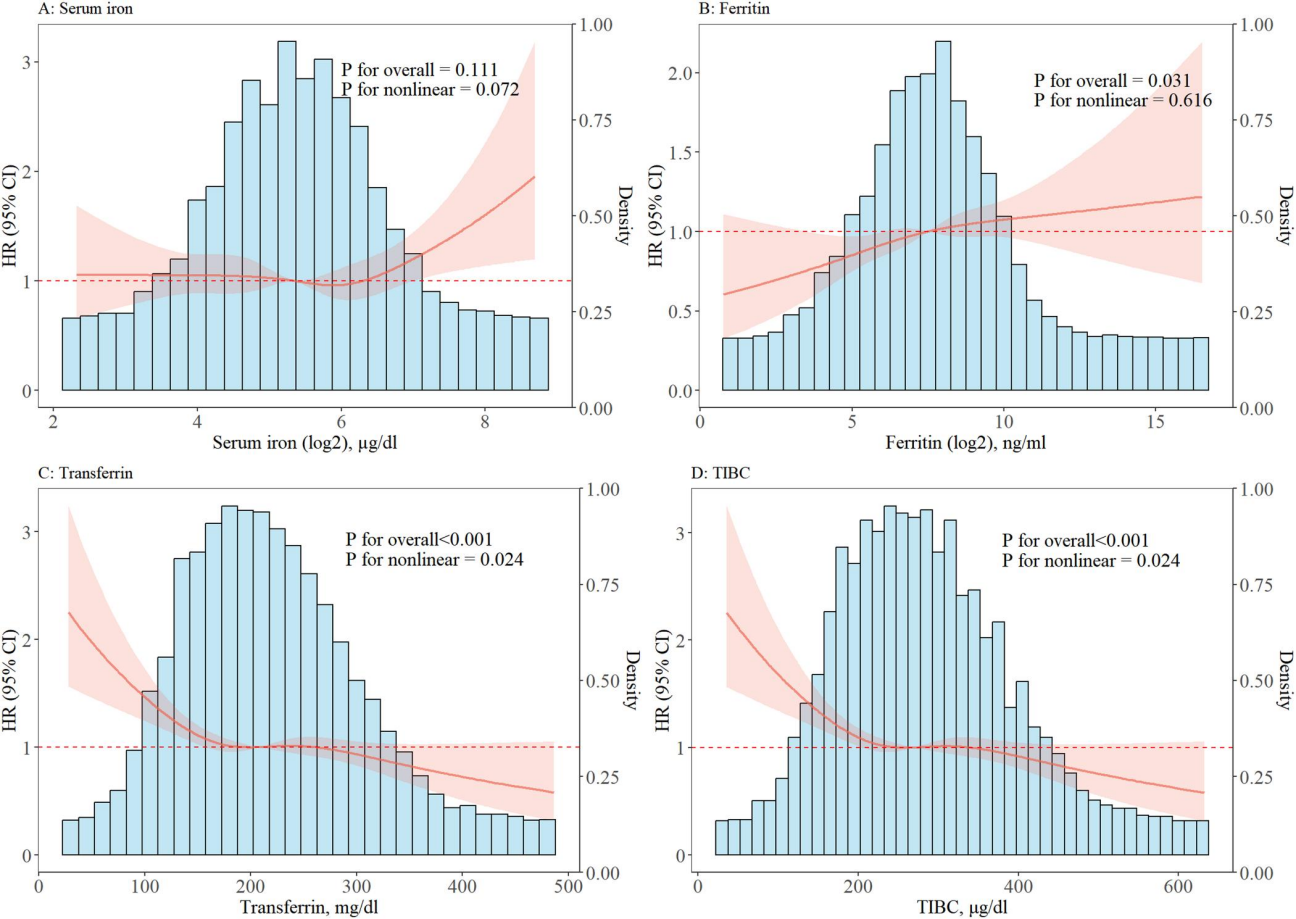
Adjusted restricted cubic spline curves for iron homeostasis related indicators and 1‐year mortality. TIBC, total iron‐binding capacity.

#### 4.2.4 Subgroup Analysis

Subgroup analyses were performed according to gender, age, race/ethnicity, diabetes, hypertension, congestive heart failure, cerebrovascular disease, new‐onset AF, SOFA, and SAPS II. Detailed results, including the analyses stratified by SOFA and SAPS II, are provided in Supporting Information S1: Tables 7−9 (Additional file 1). Selected subgroup results for ferritin (Figure 4), transferrin (Figure 5), and TIBC (Figure 6) are presented graphically.

**Figure 4:**
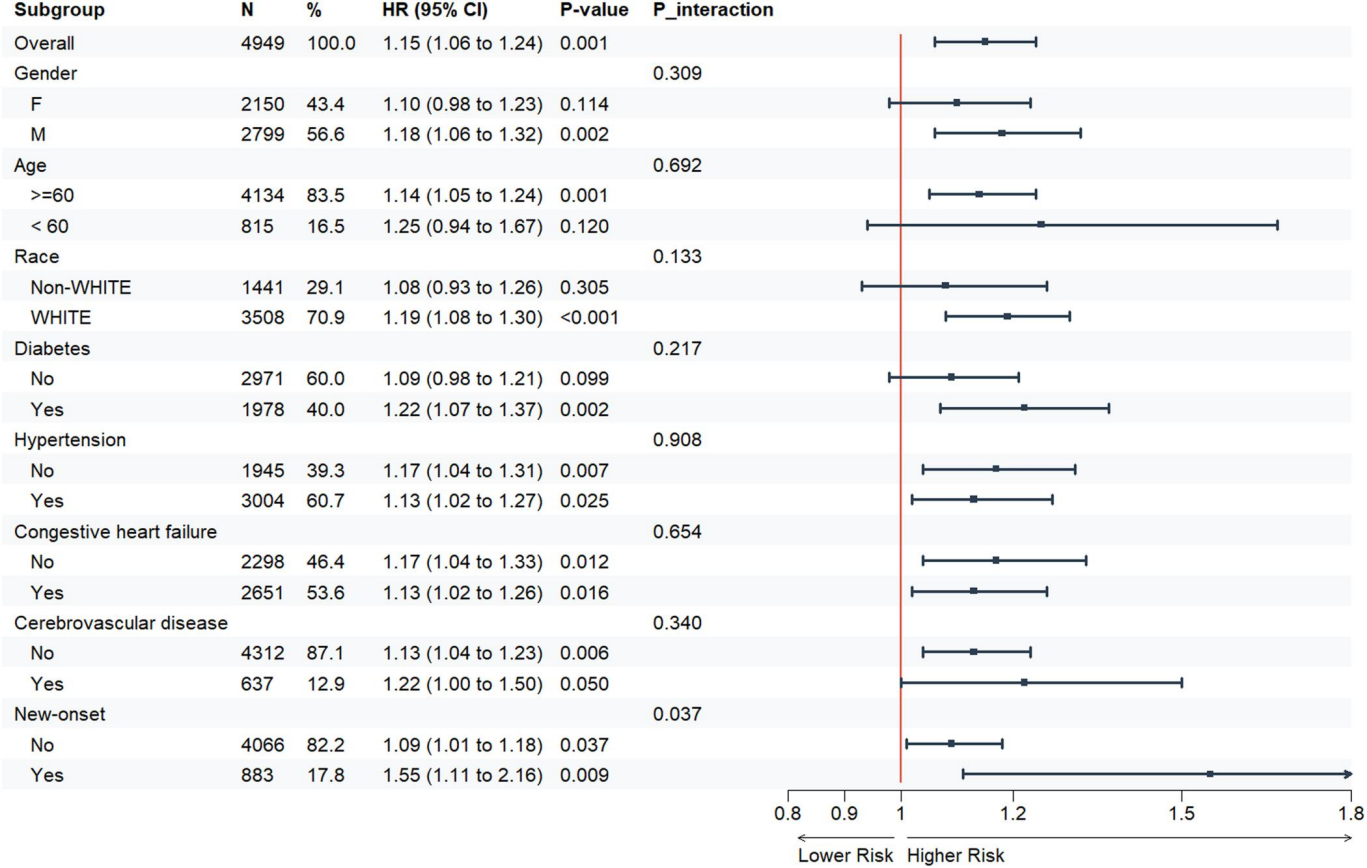
Forest plots of subgroup analyses of ferritin and 1‐year all‐cause mortality.

**Figure 5:**
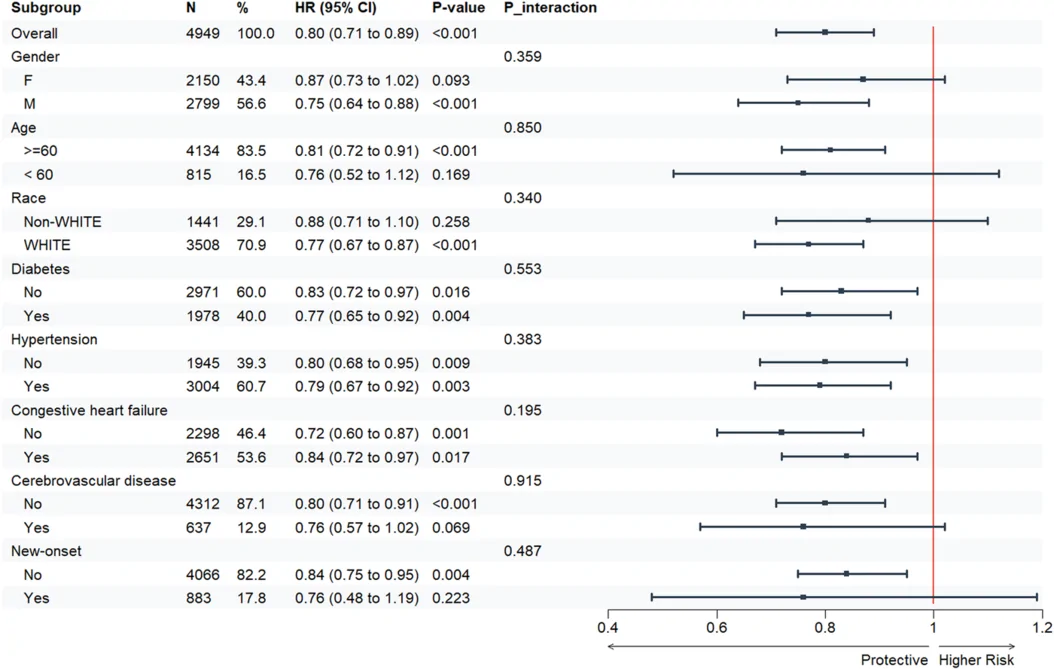
Forest plots of subgroup analyses of transferrin and 1‐year all‐cause mortality.

**Figure 6:**
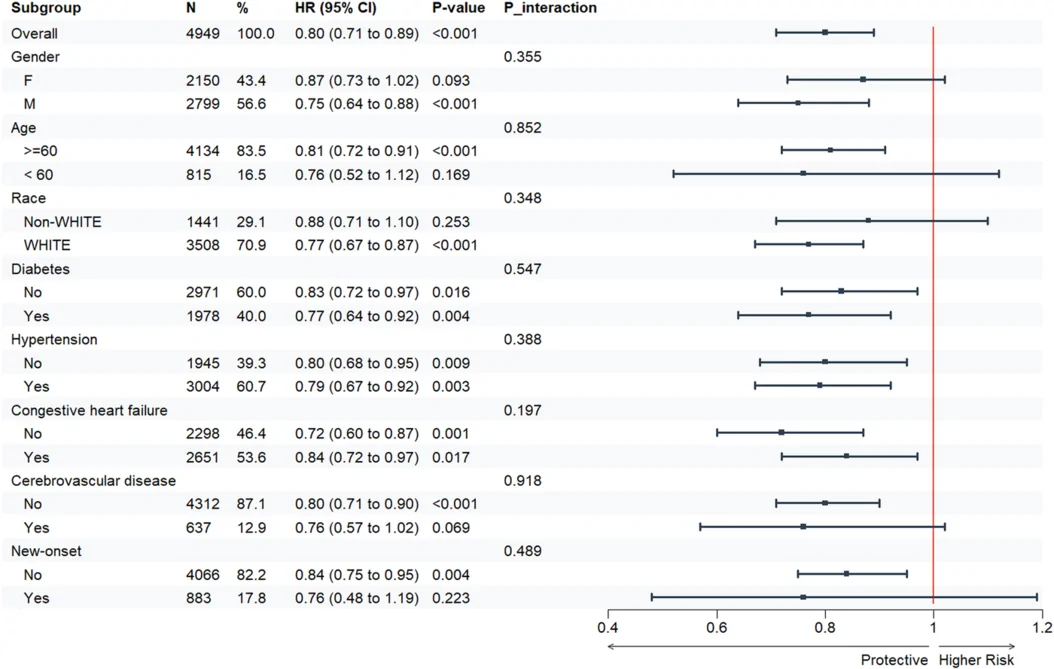
Forest plots of subgroup analyses of TIBC and 1‐year all‐cause mortality.

An interaction with new‐onset AF was observed for the association between ferritin and 1‐year mortality. No corresponding interaction was identified for transferrin or TIBC. The association between ferritin and 1‐year mortality also differed by SAPS II and SOFA levels. For transferrin and TIBC, significant interactions were observed with SAPS II.

## 5 Discussion

Iron homeostasis‐related indicators have been shown to be closely related to all‐cause mortality in critically ill AF patients. Previous studies have demonstrated that iron metabolism plays a crucial role in cardiovascular diseases [16]. Abnormal myocardial iron concentrations may contribute to AF through cardiac ion channel dysfunction [17], oxidative stress [18], and inflammatory cascades [19]. A Mendelian randomization study further suggested that increased iron status may increase the risk of AF [20]. These findings support a relation between iron homeostasis and AF pathogenesis.

In the present study, a linear relationship was observed between the log‐transformed ferritin levels and 1‐year mortality among critically ill AF patients. Serum iron was not independently associated with 1‐year mortality after multivariable adjustment. This finding is broadly consistent with previous studies reporting no significant association between iron deficiency anemia and mortality risk in patients with AF [21]. Compared with serum iron, ferritin serves as a more sensitive and accurate biomarker of intracellular iron reserves, and plays an important role in iron metabolism and iron homeostasis [22, 23]. Elevated ferritin is commonly observed in inflammatory states and may reflect ongoing inflammation [24, 25]. In addition, ferritin stratified by an iron overload threshold (SF > 1000 ng/ml) was associated with prognosis in univariate analysis, but the association was no longer statistically significant in the fully adjusted model. This result suggests that marked hyperferritinemia may partly reflect disease severity and systemic inflammation. An analysis of hospitalized patients similarly found that elevated baseline ferritin was associated with long‐term mortality, with inflammation considered a possible contributing factor [26]. This association may reflect the chronic damage of persistent inflammation to the cardiovascular system, including oxidative stress, endothelial dysfunction, and myocardial remodeling [27, 28, 29]. In some studies, it has been regarded as an indicator for assessing disease severity and prognosis [30, 31, 32].

Higher transferrin and TIBC levels were associated with lower 1‐year mortality in our cohort. Transferrin is an iron‐binding protein that transports iron into cells by binding to transferrin receptors on the cell surface [33]. In the evaluation of iron metabolism, TIBC and transferrin concentration measurements are both used to reflect the iron transport capacity, and previous studies have reported a close association between them [34, 35]. Changes in transferrin levels are influenced by acute or chronic inflammation [36]. As transferrin is synthesized in the liver, its level is also related to protein nutritional status. For instance, in patients with Protein‐Energy Malnutrition (PEM), transferrin levels decrease due to impaired hepatic protein synthesis [37, 38]. In critically ill patients, malnutrition and inflammation frequently coexist, rendering the interpretation of transferrin levels particularly complex. Decreased transferrin levels may reflect both nutritional deficiency and inflammation, which are associated with adverse clinical outcomes [39, 40, 41]. Studies have suggested that transferrin regulates iron metabolism to preserve cellular viability and may have therapeutic potential in cardiovascular diseases [42]. Transferrin exerts a protective effect by sequestering free iron in tissues and cardiomyocytes to mitigate iron‐induced ROS‐mediated injury. Transferrin supplementation has been explored in other conditions characterized by iron toxicity [43]. However, the clinical value of this intervention requires further evaluation in prospective studies and randomized controlled trials.

Alterations in iron homeostasis are intricately linked to the prognosis of AF. First, cellular iron can directly interact with and disrupt various cardiac ion channels, thereby interfering with cardiac electrophysiological conduction [17, 44]. Second, excess iron can promote the Fenton reaction and the generation of reactive oxygen species, contributing to oxidative cellular injury [45]. Huang et al. similarly reported that selected iron‐related biomarkers were associated with increased mortality in critically ill patients with AF, potentially through oxidative stress and inflammatory responses related to iron overload [46]. Disturbances in iron homeostasis may also occur alongside inflammation and malnutrition [47, 48, 49]. These conditions frequently coexist and are associated with adverse outcomes [50, 51]. Inflammation and poor nutritional status may reinforce one another and contribute to atrial remodeling and fibrosis, which may in turn be associated with recurrent arrhythmia and mortality [50, 52, 53].

Notably, the 1‐year mortality rate observed in this study was higher than that reported in general AF populations. In the ICU, patients with AF have significantly higher in‐hospital and out‐of‐hospital mortality rates compared to ICU patients without AF [54]. This difference may be related to the inclusion of critically ill ICU patients with a greater burden of comorbidities and acute organ dysfunction [55]. The high 1‐year mortality observed in this ICU cohort highlights the potential relevance of routinely available iron homeostasis‐related measurements for prognostic assessments. Ferritin, transferrin, and TIBC are readily available laboratory parameters, and their associations with mortality suggest that they may provide useful prognostic information in critically ill patients with AF. Further prognostic studies are needed to determine whether these markers offer additional prognostic value beyond established clinical risk factors.

There are several limitations in the present study. First, because this retrospective investigation relied on observational data from MIMIC‐IV, causal relationships could not be established. Secondly, the single‐center design and relatively short follow‐up may limit the generalizability and precision of our findings. Validation in larger prospective, multicenter cohorts is therefore needed. Thirdly, key indicators such as left ventricular ejection fraction (LVEF) were not included in our analysis. LVEF is an essential indicator for risk stratification and treatment guidance in AF patients, and its absence represents an important methodological limitation of this study. Therefore, the relevant findings should be interpreted with caution. Other indicators (e.g., CRP and albumin) also had insufficient available data, and the ability to distinguish AF subtypes (paroxysmal, persistent, and long‐standing persistent AF) was limited. Furthermore, because iron homeostasis measurements and hemoglobin were available only at ICU admission, our analysis could not capture changes in iron status and bleeding/transfusion events during the course of critical illness or determine whether such changes were associated with subsequent outcomes. Serial assessments of these parameters may provide a more complete characterization of iron homeostasis and its relationship with long‐term prognosis.

## 6 Conclusions

Among critically ill patients with AF, higher ferritin levels and lower transferrin and TIBC levels were independently associated with greater 1‐year all‐cause mortality, whereas serum iron was not significantly associated with mortality after multivariable adjustment. The observed associations suggest a relationship between iron homeostasis and long‐term outcomes in this population. Prospective studies with serial measurements and external validation are needed to assess whether these parameters provide additional prognostic information beyond established clinical risk factors.

## Author Contributions

Mingze Wu and Juan Hua analyzed and interpreted critically ill atrial fibrillation patient data and were the main contributors to writing the manuscript. Yao Zhou and Yu Wang collected the data. Yu Zhang and Lin Zhang participated in editing the manuscript. Qinmei Xiong aided in the conception and design of this study. All authors read and approved the final manuscript.

## Conflicts of Interest

The authors declare no conflicts of interest.

## Supporting information


Supporting File 1

Supporting File 2

## Data Availability

This study utilized data from the MIMIC‐IV (version 3.1) database. Formal applications for access can be submitted via the official website (https://physionet.org/content/mimiciv/3.1/). The first author obtained approval (certification number: 65951130). The data are available from PhysioNet to credentialed users who meet the access requirements and sign the data use agreement.
