# Supplementary material for: Association of Iron Homeostasis With One‐Year All‐Cause Mortality Among Intensive Care Unit Patients with Atrial Fibrillation: A Retrospective Study
Source: Clin Cardiol. 2026 Oct 14;49(10):e70494. doi: 10.1002/clc.70494 (PMC13664674; doi:10.1002/clc.70494)
Supplement: Supplementary file 1 [file CLC-49-e70494-s001.docx]

Supplementary Table 1. Baseline characteristics of patients with atrial fibrillation

| **Characteristic** | **N** | **Overall** N = 4,949 | **Non-survivors** N = 1,375 | **Survivors** N = 3,574 | **p-value** |
| --- | --- | --- | --- | --- | --- |
| Age | 4,949 | 73.00 (64.00, 81.00) | 77.00 (70.00, 84.00) | 71.00 (62.00, 79.00) | <0.001 |
| Gender, n (%) | 4,949 |  |  |  | 0.122 |
| F |  | 2,150 (43) | 622 (45) | 1,528 (43) |  |
| M |  | 2,799 (57) | 753 (55) | 2,046 (57) |  |
| Race, n (%) | 4,949 |  |  |  | 0.096 |
| Non-WHITE |  | 1,441 (29) | 376 (27) | 1,065 (30) |  |
| WHITE |  | 3,508 (71) | 999 (73) | 2,509 (70) |  |
| Weight, kg | 4,949 | 79.00 (66.40, 95.15) | 74.20 (62.30, 89.40) | 81.35 (68.00, 97.00) | <0.001 |
| Hypertension, n (%) | 4,949 | 3,004 (61) | 687 (50) | 2,317 (65) | <0.001 |
| Diabetes, n (%) | 4,949 | 1,978 (40) | 572 (42) | 1,406 (39) | 0.155 |
| Chronic pulmonary disease, n (%) | 4,949 | 1,417 (29) | 423 (31) | 994 (28) | 0.043 |
| Myocardial infarct, n (%) | 4,949 | 1,082 (22) | 371 (27) | 711 (20) | <0.001 |
| Congestive heart failure, n (%) | 4,949 | 2,651 (54) | 813 (59) | 1,838 (51) | <0.001 |
| Peripheral vascular disease, n (%) | 4,949 | 821 (17) | 251 (18) | 570 (16) | 0.056 |
| Cerebrovascular disease, n (%) | 4,949 | 637 (13) | 219 (16) | 418 (12) | <0.001 |
| Dementia, n (%) | 4,949 | 248 (5.0) | 117 (8.5) | 131 (3.7) | <0.001 |
| SBP, mmHg | 4,949 | 114.40 (105.64, 126.33) | 111.77 (103.15, 123.48) | 115.13 (106.42, 127.22) | <0.001 |
| DBP, mmHg | 4,949 | 59.96 (53.70, 67.60) | 59.13 (52.31, 67.08) | 60.35 (54.16, 67.92) | <0.001 |
| MBP, mmHg | 4,949 | 75.44 (69.77, 82.72) | 74.12 (68.32, 81.33) | 75.96 (70.32, 83.16) | <0.001 |
| Heart rate, bpm | 4,949 | 82.31 (72.40, 93.74) | 83.60 (72.56, 95.29) | 81.92 (72.30, 93.21) | 0.018 |
| Respiratory rate, bpm | 4,949 | 19.02 (16.92, 21.74) | 19.75 (17.42, 22.68) | 18.76 (16.76, 21.36) | <0.001 |
| Temperature, ℃ | 4,949 | 36.74 (36.52, 36.99) | 36.70 (36.47, 36.93) | 36.76 (36.54, 37.01) | <0.001 |
| WBC, 10^9^/L | 4,949 | 10.66 (7.85, 14.50) | 10.95 (7.85, 15.30) | 10.55 (7.85, 14.23) | 0.009 |
| Hemoglobin, g/dL | 4,949 | 9.85 (8.63, 11.30) | 9.40 (8.37, 10.83) | 10.03 (8.75, 11.55) | <0.001 |
| Platelets, 10^9^/L | 4,949 | 194.00 (144.00, 263.33) | 200.00 (144.00, 277.00) | 192.50 (144.00, 259.00) | 0.057 |
| BUN, mg/dL | 4,949 | 27.50 (17.67, 45.40) | 36.67 (24.00, 59.67) | 24.67 (16.50, 39.67) | <0.001 |
| Creatinine, mg/dL | 4,949 | 1.27 (0.90, 2.07) | 1.60 (1.03, 2.74) | 1.18 (0.85, 1.83) | <0.001 |
| Glucose, mg/dL | 4,949 | 129.50 (108.00, 161.00) | 132.67 (109.00, 169.50) | 128.00 (108.00, 158.25) | 0.005 |
| Chloride, mEq/L | 4,949 | 103.00 (98.50, 107.00) | 101.60 (97.00, 106.00) | 103.50 (99.00, 107.00) | <0.001 |
| Sodium, mEq/L | 4,949 | 138.00 (135.33, 140.67) | 138.00 (135.00, 141.00) | 138.00 (135.50, 140.50) | 0.684 |
| Potassium, mEq/L | 4,949 | 4.23 (3.90, 4.65) | 4.30 (3.90, 4.73) | 4.23 (3.90, 4.60) | 0.072 |
| INR | 4,949 | 1.35 (1.20, 1.73) | 1.43 (1.20, 2.05) | 1.33 (1.20, 1.65) | <0.001 |
| PT, s | 4,949 | 14.90 (13.10, 19.07) | 15.78 (13.50, 22.00) | 14.70 (13.00, 18.10) | <0.001 |
| PTT, s | 4,949 | 33.40 (28.60, 43.45) | 34.80 (29.30, 46.13) | 32.90 (28.43, 42.43) | <0.001 |
| SpO2, % | 4,949 | 96.96 (95.61, 98.29) | 96.86 (95.44, 98.32) | 97.00 (95.65, 98.27) | 0.101 |
| Anion gap, mmol/L | 4,949 | 14.00 (12.00, 17.00) | 15.00 (12.60, 18.00) | 14.00 (11.67, 16.33) | <0.001 |
| Bicarbonate, mmol/L | 4,949 | 23.25 (20.67, 26.00) | 23.00 (20.00, 26.50) | 23.33 (21.00, 25.80) | 0.389 |
| GCS | 4,949 | 15.00 (14.00, 15.00) | 15.00 (13.00, 15.00) | 15.00 (14.00, 15.00) | <0.001 |
| SAPS II | 4,949 | 38.00 (31.00, 46.00) | 44.00 (36.00, 52.00) | 37.00 (30.00, 44.00) | <0.001 |
| SOFA | 4,949 | 5.00 (3.00, 7.00) | 5.00 (3.00, 8.00) | 4.00 (2.00, 7.00) | <0.001 |
| Aspirin, n (%) | 4,949 | 2,777 (56) | 710 (52) | 2,067 (58) | <0.001 |
| Clopidogrel, n (%) | 4,949 | 568 (11) | 162 (12) | 406 (11) | 0.713 |
| Beta blocker, n (%) | 4,949 | 3,968 (80) | 1,020 (74) | 2,948 (82) | <0.001 |
| ACEI/ARB, n (%) | 4,949 | 1,680 (34) | 337 (25) | 1,343 (38) | <0.001 |
| CCB, n (%) | 4,949 | 1,718 (35) | 411 (30) | 1,307 (37) | <0.001 |
| Digoxin, n (%) | 4,949 | 522 (11) | 194 (14) | 328 (9.2) | <0.001 |
| Loop diuretic, n (%) | 4,949 | 3,585 (72) | 1,023 (74) | 2,562 (72) | 0.060 |
| MRA, n (%) | 4,949 | 339 (6.8) | 77 (5.6) | 262 (7.3) | 0.036 |
| Thiazide, n (%) | 4,949 | 627 (13) | 203 (15) | 424 (12) | 0.007 |
| Amiodarone, n (%) | 4,949 | 1,456 (29) | 403 (29) | 1,053 (29) | 0.943 |
| Insulin, n (%) | 4,949 | 3,297 (67) | 891 (65) | 2,406 (67) | 0.099 |
| Statin, n (%) | 4,949 | 2,616 (53) | 669 (49) | 1,947 (54) | <0.001 |
| NOACs, n (%) | 4,949 | 900 (18) | 241 (18) | 659 (18) | 0.482 |
| Heparin, n (%) | 4,949 | 4,043 (82) | 1,173 (85) | 2,870 (80) | <0.001 |
| Warfarin, n (%) | 4,949 | 1,755 (35) | 403 (29) | 1,352 (38) | <0.001 |
| Ferritin, ng/ml | 4,949 | 182.00 (74.00, 427.00) | 218.00 (93.00, 513.00) | 170.00 (68.00, 390.00) | <0.001 |
| Iron, µg/dl | 4,949 | 41.00 (26.00, 64.00) | 38.00 (25.00, 63.00) | 41.50 (26.00, 64.00) | 0.084 |
| TIBC, µg/dl | 4,949 | 270.00 (208.00, 338.00) | 250.00 (191.00, 320.00) | 277.00 (215.00, 345.00) | <0.001 |
| Transferrin, mg/dl | 4,949 | 208.00 (160.00, 260.00) | 192.00 (147.00, 246.00) | 213.00 (165.00, 265.00) | <0.001 |
| LOS ICU, days | 4,949 | 2.83 (1.70, 5.40) | 3.46 (1.93, 7.67) | 2.58 (1.60, 4.83) | <0.001 |
| LOS hospital, days | 4,949 | 9.66 (5.99, 16.65) | 11.84 (6.60, 20.94) | 8.99 (5.83, 15.09) | <0.001 |
| 30 days death, n (%) | 4,949 | 529 (11) | 529 (38) | 0 (0) |  |
| 60 days death, n (%) | 4,949 | 741 (15) | 741 (54) | 0 (0) |  |
| 90 days death, n (%) | 4,949 | 874 (18) | 874 (64) | 0 (0) |  |
| 180 days death, n (%) | 4,949 | 1,104 (22) | 1,104 (80) | 0 (0) |  |

Data are presented as numbers and percentage or median and interquartile range.

Abbreviations: SBP, systolic blood pressure; DBP, diastolic blood pressure; MBP, mean blood pressure; SPO2, pulse blood oxygen saturation; WBC, white blood cell; BUN, blood urea nitrogen; INR, international normalized ratio; PT, prothrombin time; PTT, partial thromboplastin time; GCS, Glasgow coma scale; SAPS II, simplified acute physiology score; SOFA, sequential organ failure assessment; NOACs, Non-Vitamin K Antagonist Oral Anticoagulants; TIBC, total iron binding capacity; ICU, intensive care unit; LOS, Length of stay;

Note: Mortality at 30, 60, 90, and 180 days represents cumulative mortality by the corresponding time points after enrollment.

SI conversion factors: to convert ferritin to μg/l, multiply by 1; serum iron to μmol/l, by 0.179; total iron binding capacity to μmol/l, by 0.179, transferrin to μmol/l, by 0.123.

Supplementary Table 2. Cox proportional hazard ratios (HR) for one-year all-cause mortality according to quartiles of iron homeostasis

| Ferritin | Model 1 | | | Model 2 | | | Model 3 | | |
| --- | --- | --- | --- | --- | --- | --- | --- | --- | --- |
|  | HR | 95% CI | P-value | HR | 95% CI | P-value | HR | 95% CI | P-value |
| Quartile | | | | | | | | | |
| Q1 | - | - | - | - | - | - | - | - | - |
| Q2 | 1.297 | 1.106-1.521 | P=0.001 | 1.325 | 1.129-1.554 | P<0.001 | 1.237 | 1.053-1.455 | P=0.010 |
| Q3 | 1.361 | 1.162-1.594 | P<0.001 | 1.432 | 1.222-1.679 | P<0.001 | 1.258 | 1.069-1.480 | P=0.006 |
| Q4 | 1.589 | 1.362-1.853 | P<0.001 | 1.823 | 1.559-2.132 | P<0.001 | 1.277 | 1.083-1.506 | P=0.004 |
|  | | | | | | | | | |
| Iron | Model 1 | | | Model 2 | | | Model 3 | | |
|  | HR | 95% CI | P-value | HR | 95% CI | P-value | HR | 95% CI | P-value |
| Quartile | | | | | | | | | |
| Q1 | - | - | - | - | - | - | - | - | - |
| Q2 | 1.039 | 0.898-1.203 | P=0.604 | 1.016 | 0.878-1.176 | P=0.827 | 1.046 | 0.902-1.212 | P=0.555 |
| Q3 | 0.857 | 0.735-0.998 | P=0.048 | 0.898 | 0.770-1.046 | P=0.167 | 0.919 | 0.786-1.074 | P=0.288 |
| Q4 | 0.938 | 0.808-1.089 | P=0.398 | 1.013 | 0.872-1.176 | P=0.870 | 1.067 | 0.914-1.245 | P=0.413 |
|  | | | | | | | | | |
| Transferrin | Model 1 | | | Model 2 | | | Model 3 | | |
|  | HR | 95% CI | P-value | HR | 95% CI | P-value | HR | 95% CI | P-value |
| Quartile | | | | | | | | | |
| Q1 | - | - | - | - | - | - | - | - | - |
| Q2 | 0.779 | 0.677-0.896 | P<0.001 | 0.749 | 0.651-0.862 | P<0.001 | 0.838 | 0.726-0.967 | P=0.015 |
| Q3 | 0.666 | 0.576-0.770 | P<0.001 | 0.663 | 0.574-0.767 | P<0.001 | 0.819 | 0.703-0.954 | P=0.010 |
| Q4 | 0.560 | 0.481-0.652 | P<0.001 | 0.579 | 0.497-0.675 | P<0.001 | 0.732 | 0.622-0.861 | P<0.001 |
|  | | | | | | | | | |
| TIBC | Model 1 | | | Model 2 | | | Model 3 | | |
|  | HR | 95% CI | P-value | HR | 95% CI | P-value | HR | 95% CI | P-value |
| Quartile | | | | | | | | | |
| Q1 | - | - | - | - | - | - | - | - | - |
| Q2 | 0.780 | 0.678-0.897 | P<0.001 | 0.750 | 0.652-0.863 | P<0.001 | 0.838 | 0.726-0.967 | P=0.016 |
| Q3 | 0.665 | 0.575-0.769 | P<0.001 | 0.662 | 0.573-0.766 | P<0.001 | 0.818 | 0.702-0.953 | P=0.010 |
| Q4 | 0.561 | 0.482-0.653 | P<0.001 | 0.579 | 0.497-0.675 | P<0.001 | 0.732 | 0.622-0.861 | P<0.001 |

CI: confidence interval, HR: hazard ratio.

Model 1: Unadjusted model.

Model 2: adjusted for gender, age, race.

Model 3: adjusted for gender, age, race, weight, diabetes, hypertension, myocardial infarct, congestive heart failure, cerebrovascular disease, dementia, rheumatic disease, systolic blood pressure, diastolic blood pressure, heart rate, resp rate, temperature, spo2, white blood count, hemoglobin, platelets, anion gap, bun, creatinine, glucose, calcium, chloride, potassium, INR, GCS, SAPS II, SOFA, Aspirin, Clopidogrel, Beta blocker, ACEI/ARB, CCB, Digoxin, Amiodarone, Statin, NOACs, Heparin, Warfarin.

Supplementary Table 3. Bonferroni and FDR Corrections for Multiple Comparisons in the Final Multivariable Model

| Ferritin | | Model 3 | | |
| --- | --- | --- | --- | --- |
|  |  | Raw P-value | Bonferroni | FDR‑BH |
| Q1 | | - | - | - |
| Q2 | | P=0.009878 | P=0.029634 | P=0.009878 |
| Q3 | | P=0.005643 | P=0.016929 | P=0.008465 |
| Q4 | | P=0.003607 | P=0.010821 | P=0.008465 |
|  |  |  |  |  |
| Iron | | Model 3 | | |
|  |  | Raw P-value | Bonferroni | FDR‑BH |
| Q1 | | - | - | - |
| Q2 | | P=0.555491 | P=1.000 | P=0.555491 |
| Q3 | | P=0.288242 | P=0.864726 | P=0.555491 |
| Q4 | | P=0.412525 | P=1.000 | P=0.555491 |
|  |  |  |  |  |
| Transferrin | | Model 3 | | |
|  |  | Raw P-value | Bonferroni | FDR‑BH |
| Q1 | | - | - | - |
| Q2 | | P=0.015337 | P=0.046011 | P=0.015337 |
| Q3 | | P=0.010271 | P=0.030813 | P=0.015337 |
| Q4 | | P=0.000169 | P=0.000507 | P=0.000507 |
|  |  |  |  |  |
| TIBC | | Model 3 | | |
|  |  | Raw P-value | Bonferroni | FDR‑BH |
| Q1 | | - | - | - |
| Q2 | | P=0.015764 | P=0.047292 | P=0.015764 |
| Q3 | | P=0.009739 | P=0.029217 | P=0.014609 |
| Q4 | | P=0.000173 | P=0.000519 | P=0.000519 |

Supplementary Table 4. Cox proportional hazard ratios (HR) for one-year all-cause mortality for continuous iron homeostasis and by median of iron homeostasis

|  | Model 1 | | | Model 2 | | | Model 3 | | |
| --- | --- | --- | --- | --- | --- | --- | --- | --- | --- |
|  | HR | 95% CI | P-value | HR | 95% CI | P-value | HR | 95% CI | P-value |
| Ferritin（log2) | 1.102 | 1.071-1.133 | P<0.001 | 1.131 | 1.099-1.164 | P<0.001 | 1.044 | 1.014-1.076 | P=0.004 |
| Median | | | | | | | | | |
| Q1 | - | - | - | - | - | - | - | - | - |
| Q2 | 1.282 | 1.152-1.452 | P<0.001 | 1.384 | 1.243-1.541 | P<0.001 | 1.125 | 1.005-1.259 | P=0.04 |
|  | | | | | | | | | |
|  | Model 1 | | | Model 2 | | | Model 3 | | |
|  | HR | 95% CI | P-value | HR | 95% CI | P-value | HR | 95% CI | P-value |
| Serum Iron（log2) | 0.962 | 0.909-1.018 | P=0.179 | 0.994 | 0.938-1.053 | P=0.838 | 1.018 | 0.959-1.080 | P=0.560 |
| Median | | | | | | | | | |
| Q1 | - | - | - | - | - | - | - | - | - |
| Q2 | 0.868 | 0.781-0.965 | P=0.009 | 0.933 | 0.839-1.038 | P=0.202 | 0.958 | 0.859-1.069 | P=0.447 |
|  | | | | | | | | | |
|  | Model 1 | | | Model 2 | | | Model 3 | | |
|  | HR | 95% CI | P-value | HR | 95% CI | P-value | HR | 95% CI | P-value |
| Transferrin | 0.997 | 0.996-0.997 | P<0.001 | 0.997 | 0.996-0.997 | P<0.001 | 0.998 | 0.997-0.999 | P<0.001 |
| Median | | | | | | | | | |
| Q1 | - | - | - | - | - | - | - | - | - |
| Q2 | 0.692 | 0.622-0.771 | P<0.001 | 0.717 | 0.644-0.799 | P<0.001 | 0.856 | 0.763-0.961 | P=0.008 |
|  | | | | | | | | | |
|  | Model 1 | | | Model 2 | | | Model 3 | | |
|  | HR | 95% CI | P-value | HR | 95% CI | P-value | HR | 95% CI | P-value |
| TIBC | 0.997 | 0.997-0.998 | P<0.001 | 0.997 | 0.997-0.998 | P<0.001 | 0.999 | 0.998-0.999 | P<0.001 |
| Median | | | | | | | | | |
| Q1 | - | - | - | - | - | - | - | - | - |
| Q2 | 0.692 | 0.621-0.770 | P<0.001 | 0.716 | 0.643-0.798 | P<0.001 | 0.855 | 0.762-0.960 | P=0.008 |

CI: confidence interval, HR: hazard ratio.

Model 1: unadjusted model.

Model 2: adjusted for gender, age, race.

Model 3: adjusted for gender, age, race, weight, diabetes, hypertension, myocardial infarct, congestive heart failure, cerebrovascular disease, dementia, rheumatic disease, systolic blood pressure, diastolic blood pressure, heart rate, resp rate, temperature, spo2, white blood count, hemoglobin, platelets, anion gap, bun, creatinine, glucose, calcium, chloride, potassium, INR, GCS, SAPS II, SOFA, Aspirin, Clopidogrel, Beta blocker, ACEI/ARB, CCB, Digoxin, Amiodarone, Statin, NOACs, Heparin, Warfarin.

Supplementary Table 5. Cox proportional hazard ratios (HR) for one-year all-cause mortality for continuous and quartile-based iron homeostasis indicators after adjustment for NLR and PLR

| Ferritin | Model | | |
| --- | --- | --- | --- |
|  | HR | 95% CI | P-value |
| Ferritin(log2), ng/mL | 1.066 | 1.027-1.106 | P<0.001 |
| Quartile | | | |
| Q1 | - | - | - |
| Q2 | 1.333 | 1.081-1.645 | P=0.007 |
| Q3 | 1.482 | 1.203-1.827 | P<0.001 |
| Q4 | 1.431 | 1.154-1.774 | P=0.001 |
|  | | | |
| Iron | Model | | |
|  | HR | 95% CI | P-value |
| Serum Iron(log2), μg/dL | 1.032 | 0.960-1.110 | P=0.389 |
| Quartile | | | |
| Q1 | - | - | - |
| Q2 | 1.027 | 0.852-1.238 | P=0.779 |
| Q3 | 0.889 | 0.730-1.084 | P=0.246 |
| Q4 | 1.09 | 0.898-1.321 | P=0.384 |
|  | | | |
| Transferrin | Model | | |
|  | HR | 95% CI | P-value |
| Transferrin, mg/dL | 0.998 | 0.997-0.999 | P<0.001 |
| Quartile | | | |
| Q1 | - | - | - |
| Q2 | 0.829 | 0.694-0.991 | P=0.040 |
| Q3 | 0.77 | 0.634-0.935 | P=0.008 |
| Q4 | 0.707 | 0.573-0.873 | P=0.001 |
|  | | | |
| TIBC | Model | | |
|  | HR | 95% CI | P-value |
| TIBC, μg/dL | 0.998 | 0.997-0.999 | P<0.001 |
| Quartile | | | |
| Q1 | - | - | - |
| Q2 | 0.830 | 0.695-0.992 | P=0.041 |
| Q3 | 0.769 | 0.633-0.934 | P=0.008 |
| Q4 | 0.707 | 0.573-0.873 | P=0.001 |

CI: confidence interval, HR: hazard ratio.

Model: adjusted for gender, age, race, weight, diabetes, hypertension, myocardial infarct, congestive heart failure, cerebrovascular disease, dementia, rheumatic disease, systolic blood pressure, diastolic blood pressure, heart rate, resp rate, temperature, spo2, white blood count, hemoglobin, platelets, anion gap, bun, creatinine, glucose, calcium, chloride, potassium, INR, GCS, SAPS II, SOFA, Aspirin, Clopidogrel, Beta blocker, ACEI/ARB, CCB, Digoxin, Amiodarone, Statin, NOACs, Heparin, Warfarin, NLR(Neutrophil-to-Lymphocyte Ratio), PLR(Platelet-to-Lymphocyte Ratio).

Supplementary Table 6. The Cox proportional regression model stratified by iron overload

| Ferritin | Unadjusted Model | | | Fully adjusted Model | | |
| --- | --- | --- | --- | --- | --- | --- |
|  | HR | 95% CI | P-value | HR | 95% CI | P-value |
| Overload | | | | | | |
| No | - | - | - | - | - | - |
| Yes | 1.457 | 1.236-1.719 | P<0.001 | 1.083 | 0.910-1.288 | P=0.369 |

CI: confidence interval, HR: hazard ratio.

Fully adjusted Model: adjusted for gender, age, race, weight, diabetes, hypertension, myocardial infarct, congestive heart failure, cerebrovascular disease, dementia, rheumatic disease, systolic blood pressure, diastolic blood pressure, heart rate, resp rate, temperature, spo2, white blood count, hemoglobin, platelets, anion gap, bun, creatinine, glucose, calcium, chloride, potassium, INR, GCS, SAPS II, SOFA, Aspirin, Clopidogrel, Beta blocker, ACEI/ARB, CCB, Digoxin, Amiodarone, Statin, NOACs, Heparin, Warfarin;

Iron overload was defined as serum ferritin >1000 ng/mL.

Supplementary Table 7. Subgroup analysis of Ferritin and All-cause mortality in patients with atrial fibrillation

| Ferritin | Count | Percent | Point Estimate | Lower | Upper | P-value | P for interaction |
| --- | --- | --- | --- | --- | --- | --- | --- |
| Overall | 4949 | 100 | 1.15 | 1.06 | 1.24 | 0.001 |  |
| Gender |  |  |  |  |  |  | 0.309 |
| F | 2150 | 43.4 | 1.1 | 0.98 | 1.23 | 0.114 |  |
| M | 2799 | 56.6 | 1.18 | 1.06 | 1.32 | 0.002 |  |
| Age |  |  |  |  |  |  | 0.692 |
| ≥60 | 4134 | 83.5 | 1.14 | 1.05 | 1.24 | 0.001 |  |
| <60 | 815 | 16.5 | 1.25 | 0.94 | 1.67 | 0.12 |  |
| Race |  |  |  |  |  |  | 0.133 |
| Non-WHITE | 1441 | 29.1 | 1.08 | 0.93 | 1.26 | 0.305 |  |
| WHITE | 3508 | 70.9 | 1.19 | 1.08 | 1.3 | <0.001 |  |
| Diabetes |  |  |  |  |  |  | 0.217 |
| No | 2971 | 60 | 1.09 | 0.98 | 1.21 | 0.099 |  |
| Yes | 1978 | 40 | 1.22 | 1.07 | 1.37 | 0.002 |  |
| Hypertension |  |  |  |  |  |  | 0.908 |
| No | 1945 | 39.3 | 1.17 | 1.04 | 1.31 | 0.007 |  |
| Yes | 3004 | 60.7 | 1.13 | 1.02 | 1.27 | 0.025 |  |
| Congestive heart failure |  |  |  |  |  |  | 0.654 |
| No | 2298 | 46.4 | 1.17 | 1.04 | 1.33 | 0.012 |  |
| Yes | 2651 | 53.6 | 1.13 | 1.02 | 1.26 | 0.016 |  |
| Cerebrovascular disease |  |  |  |  |  |  | 0.34 |
| No | 4312 | 87.1 | 1.13 | 1.04 | 1.23 | 0.006 |  |
| Yes | 637 | 12.9 | 1.22 | 1 | 1.5 | 0.05 |  |
| New-onset |  |  |  |  |  |  | 0.037 |
| No | 4066 | 82.2 | 1.09 | 1.01 | 1.18 | 0.037 |  |
| Yes | 883 | 17.8 | 1.55 | 1.11 | 2.16 | 0.009 |  |
| SAPS II group |  |  |  |  |  |  | 0.001 |
| Low | 2515 | 50.8 | 1.33 | 1.16 | 1.53 | <0.001 |  |
| High | 2434 | 49.2 | 1.02 | 0.93 | 1.13 | 0.614 |  |
| SOFA group |  |  |  |  |  |  | 0.003 |
| Low | 2471 | 49.9 | 1.25 | 1.1 | 1.42 | 0.001 |  |
| High | 2478 | 50.1 | 1 | 0.9 | 1.11 | 0.979 |  |

Supplementary Table 8. Subgroup analysis of Transferrin and All-cause mortality in patients with atrial fibrillation

| Transferrin | Count | Percent | Point Estimate | Lower | Upper | P-value | P for interaction |
| --- | --- | --- | --- | --- | --- | --- | --- |
| Overall | 4949 | 100 | 0.8 | 0.71 | 0.89 | <0.001 |  |
| Gender |  |  |  |  |  |  | 0.506 |
| F | 2150 | 43.4 | 0.85 | 0.72 | 1.01 | 0.065 |  |
| M | 2799 | 56.6 | 0.76 | 0.65 | 0.89 | 0.001 |  |
| Age |  |  |  |  |  |  | 0.912 |
| ≥60 | 4134 | 83.5 | 0.8 | 0.71 | 0.9 | <0.001 |  |
| <60 | 815 | 16.5 | 0.77 | 0.53 | 1.14 | 0.192 |  |
| Race |  |  |  |  |  |  | 0.21 |
| Non-WHITE | 1441 | 29.1 | 0.9 | 0.72 | 1.12 | 0.334 |  |
| WHITE | 3508 | 70.9 | 0.76 | 0.67 | 0.87 | <0.001 |  |
| Diabetes |  |  |  |  |  |  | 0.611 |
| No | 2971 | 60 | 0.83 | 0.72 | 0.96 | 0.014 |  |
| Yes | 1978 | 40 | 0.77 | 0.64 | 0.92 | 0.003 |  |
| Hypertension |  |  |  |  |  |  | 0.559 |
| No | 1945 | 39.3 | 0.79 | 0.67 | 0.93 | 0.005 |  |
| Yes | 3004 | 60.7 | 0.8 | 0.68 | 0.94 | 0.005 |  |
| Congestive heart failure |  |  |  |  |  |  | 0.327 |
| No | 2298 | 46.4 | 0.75 | 0.62 | 0.9 | 0.002 |  |
| Yes | 2651 | 53.6 | 0.82 | 0.71 | 0.95 | 0.009 |  |
| Cerebrovascular disease |  |  |  |  |  |  | 0.806 |
| No | 4312 | 87.1 | 0.8 | 0.71 | 0.91 | <0.001 |  |
| Yes | 637 | 12.9 | 0.76 | 0.56 | 1.02 | 0.067 |  |
| New-onset |  |  |  |  |  |  | 0.497 |
| No | 4066 | 82.2 | 0.84 | 0.74 | 0.94 | 0.003 |  |
| Yes | 883 | 17.8 | 0.78 | 0.49 | 1.22 | 0.277 |  |
| SAPS II group |  |  |  |  |  |  | 0.024 |
| Low | 2515 | 50.8 | 0.71 | 0.58 | 0.86 | <0.001 |  |
| High | 2434 | 49.2 | 0.91 | 0.79 | 1.04 | 0.175 |  |
| SOFA group |  |  |  |  |  |  | 0.126 |
| Low | 2471 | 49.9 | 0.75 | 0.62 | 0.89 | 0.001 |  |
| High | 2478 | 50.1 | 0.9 | 0.78 | 1.04 | 0.161 |  |

Supplementary Table 9. Subgroup analysis of TIBC and All-cause mortality in patients with atrial fibrillation

| TIBC | Count | Percent | Point Estimate | Lower | Upper | P-value | P for interaction |
| --- | --- | --- | --- | --- | --- | --- | --- |
| Overall | 4949 | 100 | 0.8 | 0.71 | 0.89 | <0.001 |  |
| Gender |  |  |  |  |  |  | 0.499 |
| F | 2150 | 43.4 | 0.85 | 0.72 | 1.01 | 0.065 |  |
| M | 2799 | 56.6 | 0.76 | 0.65 | 0.89 | 0.001 |  |
| Age |  |  |  |  |  |  | 0.915 |
| ≥60 | 4134 | 83.5 | 0.8 | 0.71 | 0.9 | <0.001 |  |
| <60 | 815 | 16.5 | 0.77 | 0.53 | 1.14 | 0.192 |  |
| Race |  |  |  |  |  |  | 0.217 |
| Non-WHITE | 1441 | 29.1 | 0.9 | 0.72 | 1.12 | 0.326 |  |
| WHITE | 3508 | 70.9 | 0.76 | 0.67 | 0.87 | <0.001 |  |
| Diabetes |  |  |  |  |  |  | 0.601 |
| No | 2971 | 60 | 0.83 | 0.72 | 0.96 | 0.014 |  |
| Yes | 1978 | 40 | 0.77 | 0.64 | 0.92 | 0.003 |  |
| Hypertension |  |  |  |  |  |  | 0.567 |
| No | 1945 | 39.3 | 0.79 | 0.67 | 0.93 | 0.005 |  |
| Yes | 3004 | 60.7 | 0.8 | 0.68 | 0.94 | 0.005 |  |
| Congestive heart failure |  |  |  |  |  |  | 0.332 |
| No | 2298 | 46.4 | 0.75 | 0.62 | 0.9 | 0.002 |  |
| Yes | 2651 | 53.6 | 0.82 | 0.71 | 0.95 | 0.009 |  |
| Cerebrovascular disease |  |  |  |  |  |  | 0.81 |
| No | 4312 | 87.1 | 0.8 | 0.71 | 0.91 | <0.001 |  |
| Yes | 637 | 12.9 | 0.76 | 0.56 | 1.02 | 0.067 |  |
| New-onset |  |  |  |  |  |  | 0.5 |
| No | 4066 | 82.2 | 0.84 | 0.74 | 0.94 | 0.003 |  |
| Yes | 883 | 17.8 | 0.78 | 0.49 | 1.22 | 0.277 |  |
| SAPS II group |  |  |  |  |  |  | 0.024 |
| Low | 2515 | 50.8 | 0.71 | 0.58 | 0.86 | <0.001 |  |
| High | 2434 | 49.2 | 0.91 | 0.79 | 1.04 | 0.17 |  |
| SOFA group |  |  |  |  |  |  | 0.129 |
| Low | 2471 | 49.9 | 0.75 | 0.62 | 0.89 | 0.001 |  |
| High | 2478 | 50.1 | 0.9 | 0.78 | 1.04 | 0.156 |  |
