# Supplementary material for: Association of Iron Homeostasis With One‐Year All‐Cause Mortality Among Intensive Care Unit Patients with Atrial Fibrillation: A Retrospective Study
Source: Clin Cardiol. 2026 Oct 14;49(10):e70494. doi: 10.1002/clc.70494 (PMC13664674; doi:10.1002/clc.70494)
Supplement: Supplementary file 2 [file CLC-49-e70494-s002.docx]

Supplementary Figures 1. Flowchart for patient screening

Supplementary Figures 2. Relationship between iron homeostasis–related indicators at different levels and long-term mortality analyzed using the Kaplan–Meier curves

Abbreviations: TIBC, total iron binding capacity

Supplementary Figures 3. Restricted cubic spline regression analysis of iron homeostasis-related indicators with all-cause mortality

Abbreviations: TIBC, total iron binding capacity

Supplementary Figures 4. Forest plots of stratified analyses of ferritin and one-year all-cause mortality

Supplementary Figures 5. Forest plots of stratified analyses of Transferrin and one-year all-cause mortality

Supplementary Figures 6. Forest plots of stratified analyses of TIBC and one-year all-cause mortality

Supplementary Figures 7. Kaplan-Meier analysis of long-term mortality stratified by quartiles of iron homeostasis indicators
